# RACGAP1 promotes the progression and poor prognosis of lung adenocarcinoma through its effects on the cell cycle and tumor stemness

**DOI:** 10.1186/s12885-023-11761-x

**Published:** 2024-01-02

**Authors:** Yafeng Liu, Tao Han, Rui Miao, Jiawei Zhou, Jianqiang Guo, Zhi Xu, Yingru Xing, Ying Bai, Jing Wu, Dong Hu

**Affiliations:** 1 School of Medicine, Anhui University of Science and Technology, Chongren Building, No 168, Taifeng St, Huainan, 232001 P.R. China; 2 Anhui Province Engineering Laboratory of Occupational Health and Safety, Anhui University of Science and Technology, Huainan, P.R. China; 3 Key Laboratory of Industrial Dust Prevention and Control & Occupational Safety and Health of the Ministry of Education, Anhui University of Science and Technology, Huainan, P.R. China; 4 Department of Clinical Laboratory, Anhui Zhongke Gengjiu Hospital, Hefei, P.R. China

**Keywords:** LUAD, RACGAP1, Cell cycle, Tumor stemness, Prognosis

## Abstract

**Objection:**

Investigating the key genes and mechanisms that influence stemness in lung adenocarcinoma.

**Methods:**

First, consistent clustering analysis was performed on lung adenocarcinoma patients using stemness scoring to classify them. Subsequently, WGCNA was utilized to identify key modules and hub genes. Then, machine learning methods were employed to screen and identify the key genes within these modules. Lastly, functional analysis of the key genes was conducted through cell scratch assays, colony formation assays, transwell migration assays, flow cytometry cell cycle analysis, and xenograft tumor models.

**Results:**

First, two groups of patients with different stemness scores were obtained, where the high stemness score group exhibited poor prognosis and immunotherapy efficacy. Next, LASSO regression analysis and random forest regression were employed to identify genes (PBK, RACGAP1) associated with high stemness scores. RACGAP1 was significantly upregulated in the high stemness score group of lung adenocarcinoma and closely correlated with clinical pathological features, poor overall survival (OS), recurrence-free survival (RFS), and unfavorable prognosis in lung adenocarcinoma patients. Knockdown of RACGAP1 suppressed the migration, proliferation, and tumor growth of cancer cells.

**Conclusion:**

RACGAP1 not only indicates poor prognosis and limited immunotherapy benefits but also serves as a potential targeted biomarker influencing tumor stemness.

**Supplementary Information:**

The online version contains supplementary material available at 10.1186/s12885-023-11761-x.

## Introduction

The number and activity of lung adenocarcinoma (LUAD) stem cells play a crucial role in determining the survival rate of patients with this disease [1]. In addition, these stem cells possess the ability to resist the effects of radiotherapy and chemotherapy, while also playing a key role in the generation of new cancer cells, thereby promoting the malignant proliferation and metastasis of tumors [2]. Consequently, these factors have significant implications for the available treatment options and the prognosis of patients. Furthermore, cancer stem cells (CSCs) possess the unique ability to self-renew and differentiate into a diverse range of cancer cells, making them pivotal mediators in processes such as cancer metastasis, drug resistance, and cancer recurrence [3].

Currently, research on stem-like cells in lung cancer primarily focuses on exploring characteristic markers, investigating the role of these cells, and exploring differentiation treatment strategies [4, 5]. Some studies have identified targeted therapies for EGFR (epidermal growth factor receptor), which exhibits abnormal mutation activity in non-small cell lung cancer [6], such as gefitinib, erlotinib, and afatinib. These drugs have shown significant reductions in the proliferation and metastasis of lung adenocarcinoma stem cells. Immunotherapy, another critical field in lung adenocarcinoma treatment, has also gained approval for non-small cell lung cancer, including lung adenocarcinoma, with PD-1/PD-L1 inhibitors like nivolumab [7], pembrolizumab [8], atezolizumab [9], and CTLA-4 inhibitors like ipilimumab [10]. However, these therapies are not universally effective for lung adenocarcinoma. Although PD-1/PD-L1 inhibitors and CTLA-4 inhibitors may produce long-term clinical responses in certain patients, many do not benefit from treatment, and the response rates remain relatively low. Moreover, these therapies can lead to severe immune-related adverse reactions, such as autoimmune diseases [11] and neurotoxicity [12].

Stratifying lung adenocarcinoma patients to provide targeted treatments and mitigate the impact of population differences on treatment outcomes is crucial. This study utilized bioinformatics methods to classify lung adenocarcinoma patients into high stem-like and low stem-like groups, exploring the prognosis and treatment differences between the two groups to enable precise and effective treatments and improve patient survival rates. Additionally, potential key genes in the low stem-like population were functionally analyzed, providing insight and a foundation for future related research.

The classification of patients with LUAD is crucial for targeted treatment, in order to avoid the impact of treatment efficacy caused by population heterogeneity. In this study, we first utilized bioinformatics methods to divide patients with LUAD into high stemness and low stemness groups, investigating the prognosis and treatment differences between the two groups. Subsequently, considering the poorer prognosis in the low stemness group, we employed four machine learning algorithms to identify two key genes, PBK and RACGAP1. Furthermore, we conducted in vivo and in vitro experiments targeting the key gene RACGAP1. The results demonstrated that the downregulation of RACGAP1 inhibited the migration, proliferation, and tumor growth of lung cancer cells. In conclusion, our findings suggest that RACGAP1 contributes to tumor progression and unfavorable prognosis by affecting the cell cycle and tumor stemness.

## Materials and methods

### Collection and analysis of LUAD data

Download and combined a total of 1358 LUAD sample from  5 datasets, TCGA-LUAD, GSE13213, GSE26939, GSE72094, and GSE31210, along with corresponding clinical and survival annotations, from the Cancer Genome Atlas Program (TCGA) (https://www.cancer.gov/ccg/research/genome-sequencing/tcga) and the Gene Expression Omnibus (GEO) database (https://www.ncbi.nlm.nih.gov/gds). Use the Combat function in the sva R package (v3.35.2) to remove batch effects.

### Identification of stemness subtypes and calculation of stemness index (mRNAsi)

We retrieved a total of 26 gene sets related to stemness from the web-based tool: StemChecker (https://stemchecker.sysbiolab.eu/). To assess the stemness levels in each patient with LUAD, we utilized the single sample gene set enrichment analysis (ssGSEA) algorithm to quantify these gene sets and generate a stemness score. Subsequently, employing the ConsensusClusterPlus R package, we performed unsupervised clustering of LUAD patients based on the 26 stemness scores in order to identify consistent patterns. In line with the approach by Malta et al., we employed a one-class logistic regression machine learning algorithm (OCLR) to calculate mRNAsi scores (ranging from 0 to 1) for each LUAD sample. This calculation was based on pluripotent stem cell samples that were strongly correlated with stemness features and can be utilized for the prediction of cancer stemness. We compared the differences in mRNAsi scores among patients belonging to different subtypes. To predict the response to immune therapy and estimate the immune therapy response of each LUAD patient, we employed the online algorithm Tumor Immune Dysfunction and Exclusion (TIDE) available at http://tide.dfci.harvard.edu/login/.

### WGCNA screening of core gene modules and functional enrichment in C2 subtypes

The WGCNA R package (v1.68) was utilized to identify co-expression gene networks representing different subtypes of stem cells in the dataset. To construct the network, the top 75% of genes with the highest median absolute deviation (MAD) were selected. The co-expression similarity matrix was computed using Pearson correlation coefficients between any two genes. A soft thresholding power of β = 3 was applied to enhance the matrix, leading to the calculation of the weighted adjacency matrix. Subsequently, the topological overlap matrix (TOM) and the dissimilarity matrix (1-TOM) were constructed based on the adjacency matrix. Genes with high interconnectivity were clustered into different gene modules, with a minimum module size set at 30. Module eigengenes (ME) were calculated to determine the association between modules and various stem cell subtypes.

Set the hub gene parameters for specific modules as gene significance (GS), where the Pearson correlation between each gene and the C2 subtype is > 0.4, and module membership (MM), where the correlation between each gene and the module is > 0.8. Subsequently, the clusterprofiler R package (v3.14.3) was employed to conduct Gene Ontology (GO) and Kyoto Encyclopedia of Genes and Genomes (KEGG) analyses [13–15] on the hub genes within the co-expression modules.

### Machine learning-based screening for identification of key genes in the C2 subtype

We employed four machine learning algorithms, namely LASSO-logistic, LASSO-cox, Random Forest, and Random Survival Forest, to select the feature genes. LASSO analysis was performed using the “glmnet” package, and ten-fold cross-validation was implemented to prevent overfitting. Feature ranking was conducted using the “randomForest” R package, utilizing the Gini importance measure (20). Additionally, we utilized the “pROC” package to plot ROC curves and evaluate the diagnostic performance of the key genes in identifying subtypes.

### Cell culture and siRNA transfection

The normal lung epithelial cell line BEAS-2B and the lung cancer cell lines A549, H1975, and H1299 were used. All cell lines were derived from the School of Medicine of Anhui University of Science and Technology. Si reagent kits were purchased from the GenePharma company. The siRNA sequences were as follows: si-RACGAP1-1 (S: 5’-GCUGAAGCAUGCACGUAAUTT-3’, AS: 5’-AUUACGUGCAUGCUUCAGCTT-3’), si-RACGAP1-2 (S: 5’-GCUCAUGUGUGACACAUCUTT-3’, AS: 5’-AGAUGUGUCACACAUGAGCTT-3’), si-RACGAP1-3 (S: 5’-CCCUGGACCUGUAAAGAAATT-3’, AS: 5’-UUUCUUUACAGGUCCAGGGTT-3’).

### Western blot

The total cellular protein was extracted using RIPA lysis buffer (990 µl RIPA 10 µl PMSF). SDS-PAGE was employed for the separation of proteins of different molecular weights in equal amounts. Relative quantification of band intensities was performed using Adobe Photoshop. Antibodies against RACGAP1 (71KDa, 1:500), CDK2 (35KDa, 1:1000), CDK4 (34KDa, 1:1000), CDK1 (34KDa, 1:1000), NIFK (KI-67) (39KDa, 1:1000) and ACTB (42KDa, 1:1000) were obtained from ABclonal. The antibody for Nanog (42KDa, 1:1000) was purchased from Cell Signaling Technology. All blots were cut prior to hybridization with antibodies. According to the required target proteins, the membrane was cut open and incubated in different boxes to eliminate the interference of some impurity bands.

### Wound healing assay

To investigate the logarithmic growth phase, A549, and H1975 cells were digested and seeded into a 6-well plate. Once the cells covered the bottom of the plate, cell scratches were created by vertically applying a 200 µL pipette tip to each well, ensuring consistent scratch width. The cell culture medium was then aspirated, and the plate was washed three times with PBS to remove any cell debris resulting from the scratches. Serum-free culture medium was added, and photographs were taken for documentation. The culture plate was placed in an incubator and removed every 6–12 h for photography. Finally, the experimental results were analyzed based on the collected image data.

### Colony formation experiment

A549 and H1975 cells in the logarithmic growth phase were prepared as cell suspensions and seeded into a 6-well culture plate at a density of 3000 cells per well. DMEM medium was used for cell culture, with medium replacement every 3 days. After 12 days of continuous culture, the cloning cultivation was terminated. The culture medium was removed, and cells were fixed with methanol at room temperature for 20 min. Cell clones were fixed by staining with 0.1% crystal violet for 20 min. The results of clone formation were documented and counted by capturing photographs.

### Transwell migration assay

The migration ability of A549 and H1975 cells was evaluated using the Transwell assay. A 1% BSA solution was prepared in a serum-free DMEM medium, and the cells were cultured in the upper chamber of the Transwell with the 1% BSA solution. The lower chamber was filled with DMEM medium containing 10% fetal bovine serum. After incubating the cells at 37 °C for 24 h, the cells in the upper chamber were removed, while those in the lower chamber were retained. The migrated cells were fixed with methanol and stained with 0.1% crystal violet. Five random staining results were captured using a 200x magnification microscope.

### Cell flow cytometry for cell cycle analysis

To isolate cells, the sample was digested with pancreatic enzymes and prepared into a single-cell suspension. The cells were then collected by centrifugation. Subsequently, 1 mL of pre-chilled PBS was added to resuspend the cells, followed by centrifugation to remove the supernatant. Next, 1 mL of 75% ethanol was added for overnight fixation at 4 °C. After centrifugation to remove the fixation solution, the cells were resuspended in 1 mL of pre-chilled PBS, centrifuged, and the supernatant was discarded. For staining, a staining kit was used, and the working solution was prepared before use with a Rnase: PI ratio of 1:9. The cells were incubated at room temperature in the dark for 60 min, protected from light, and then analyzed using a flow cytometer. The red fluorescence was detected at an excitation wavelength of 488 nm, while the light scattering was simultaneously measured. Cell statistical analysis was performed using Modfit 5.0 software.

### Heterogeneous transplantation tumor growth

C57BL/6 male mice (6–8 weeks old, weighing 20-25 g) were obtained from Henan Skbay Biological Technology Co., Ltd. (China, Henan). The animals were maintained following institutional policies, and all studies were conducted with the approval of the Medical School of Anhui University of Science and Technology. To generate xenografts, 3 × 106 LA-4 cells were mixed with PBS and subcutaneously injected into each mouse. Tumor growth was measured using calipers, and the volume was calculated using the formula V = (a × b2)/2, where a represented the longest diameter and b represented the shortest diameter of the tumor. At the end of the experiment, the mice were killed by cervical dislocation, the tumor mass was removed and photographs were taken.

### Statistical analysis

All statistical analyses were performed using R software (version 3.6.3). Wilcoxon test was used for pairwise comparisons between two groups, while the Kruskal-Wallis test was employed for multiple group comparisons. Kaplan-Meier method and log-rank test were used for survival analysis. The optimal cut-off value for stemness-risk score was determined using the “surv_cutpoint” function from the survminer R package (version 0.4.6). A *p*-value less than 0.05 was considered statistically significant for detecting differences.

## Results

### Genotype classification based on stemness genes

**Fig. 1:**
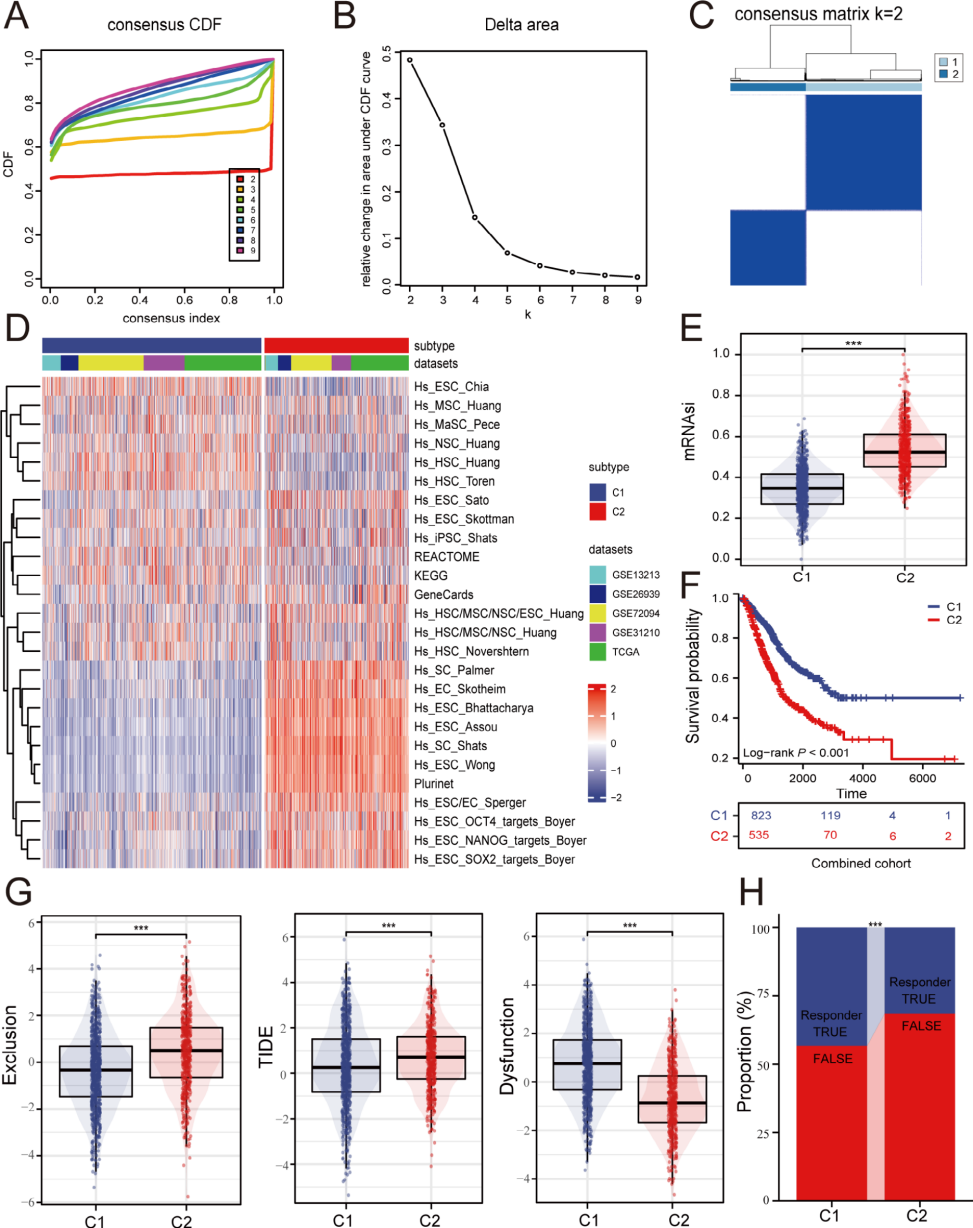
Consensus clustering identified two stemness-based subtypes. (**A-B**) Delta area curves of consensus clustering; (**C**) Heatmap depicting consensus clustering solution (k = 2) for 26 stemness scores in LUAD;(**D**) Heat map of the distribution of 26 stemness scores in different stemness subtypes;(**E**) Distribution of mRNAsi in different stemness subtypes;(**F**) Kaplan–Meier overall survival curves for patients with lung adenocarcinoma between different stemness subtypes; (**G**) The distribution of Exclusion, TIDE and Dysfunction scores in the different subtypes of stemness; (**H**) Distributions of responder and non-responder to immunotherapy predicted by the TIDE algorithm among distinct stemness clusters. (***p < 0.001; **p < 0.01; *p < 0.05; ns: not significant)

Based on the ssGSEA scores of a total of 26 gene sets, LUAD patients were classified into two distinct clusters using the Consensus Cluster Plus package for unsupervised clustering (Fig. 1A-C). Subsequently, the expression profiles of the 26 gene sets were analyzed in the two clusters, revealing a higher enrichment in the majority of gene sets in Cluster 2 (Fig. 1D). Furthermore, the expression of the gene set associated with the stemness index (mRNAsi) was examined in both clusters, confirming Cluster 2 as the high stemness group (Fig. 1E). Kaplan-Meier analysis demonstrated that LUAD patients in Cluster 2 had a worse overall survival (Fig. 1F). Additionally, the study evaluated the immune therapy response of the two subtypes using the Exclusion algorithm and TIDE algorithm, revealing a potentially lower sensitivity to immunotherapy in the C2 subtype (Fig. 1G). The immune response rate also supported these findings, showing a lower immune response rate in the C2 subtype (Fig. 1H).

### WGCNA identification of C2 cluster-related modules and hub genes

**Fig. 2:**
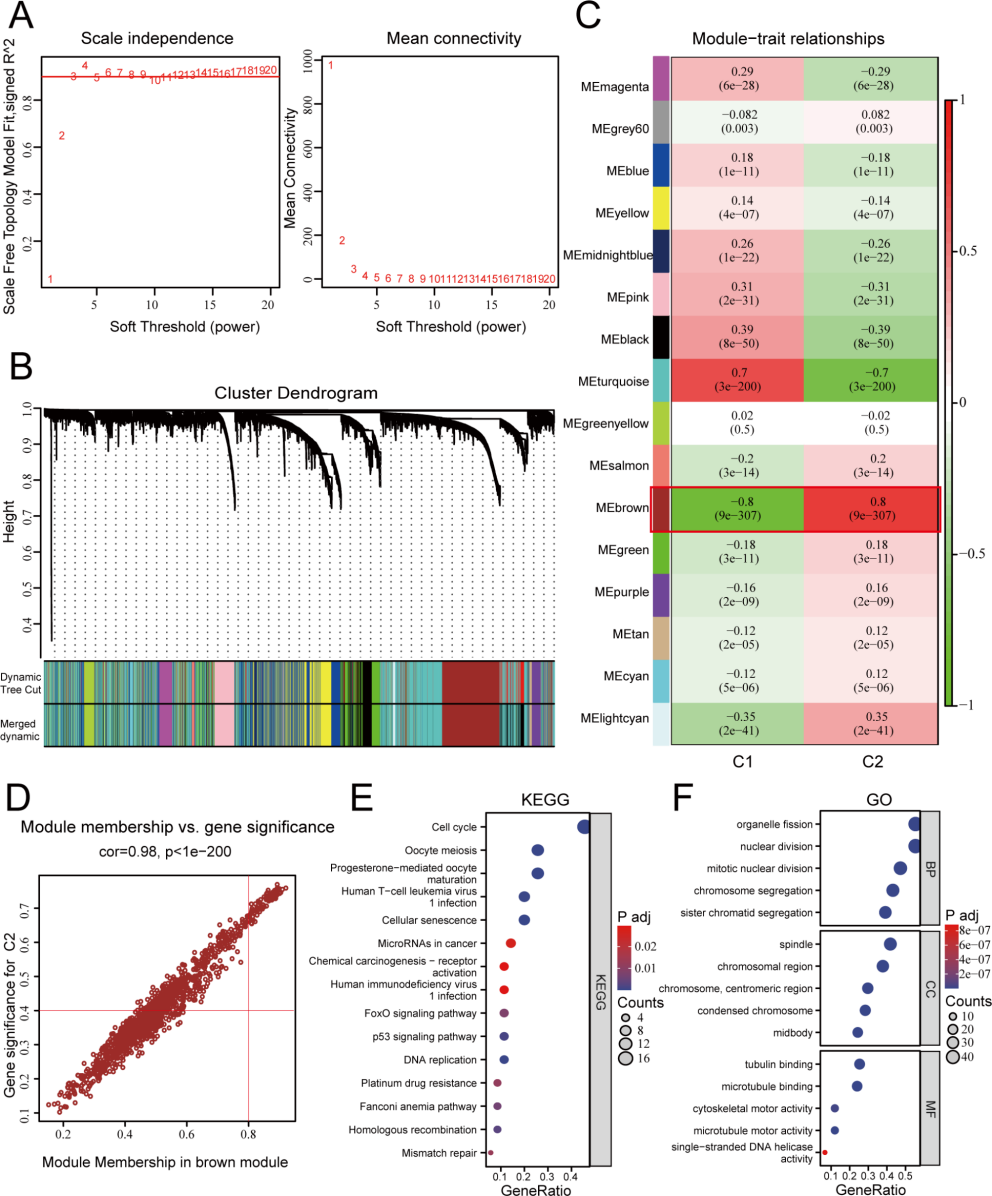
Identification of hub genes associated with stemness subtypes. (**A**) Scale independence and mean connectivity of multiple soft-thresholding powers (β) from 1 to 20; (**B**) The cluster dendrogram developed by the weighted correlation coefficients, genes with similar expression patterns were clustered into co-expression modules, and each color represents a module; (**C**) Heatmap of the correlation between module eigengenes (MEs) and clinical traits as well as stemness subtypes; (**D**) Scatter plot displaying relationship of module membership (MM) in the brown module with gene significance (GS) for Cluster C2; (**E**) KEGG analysis of Cluster2-associated module gene; (**F**) Top 15 enriched biological process (BP), cellular component (CC) and molecular function (MF) GO terms of Cluster2-associated module genes

Due to the limited benefits and poor survival rates of C2 subtype patients with LUAD in immunotherapy, WGCNA was further conducted to identify the characteristic genes of this subtype. Firstly, the optimal soft-thresholding power β was set to 3 to ensure the construction of a scale-free network (Fig. 2A). Then, the minimum gene count for each module was set to 30, and through the hierarchical clustering dendrogram, 16 modules were identified that grouped genes with similar expression patterns (Fig. 2B). Among the 16 modules, MEbrown showed the strongest positive correlation with the C2 subtype (ME = 0.8, *P* = 9e − 307), and the strongest negative correlation with the C1 subtype (ME= -0.8, *P* = 9e − 307) (Fig. 2C). Therefore, the brown module (1220 genes) was selected as the hub module, and further analysis was conducted using MM > 0.8 and GS > 0.4 as filtering criteria, resulting in 82 candidate hub genes that overlapped (Fig. 2D). In addition, to explore the biological functions of the hub genes in the MEbrown module, we performed GO and KEGG pathway enrichment analysis (Fig. 2E-F).

### Identifying key genes for C2 subtypes based on machine learning

**Fig. 3:**
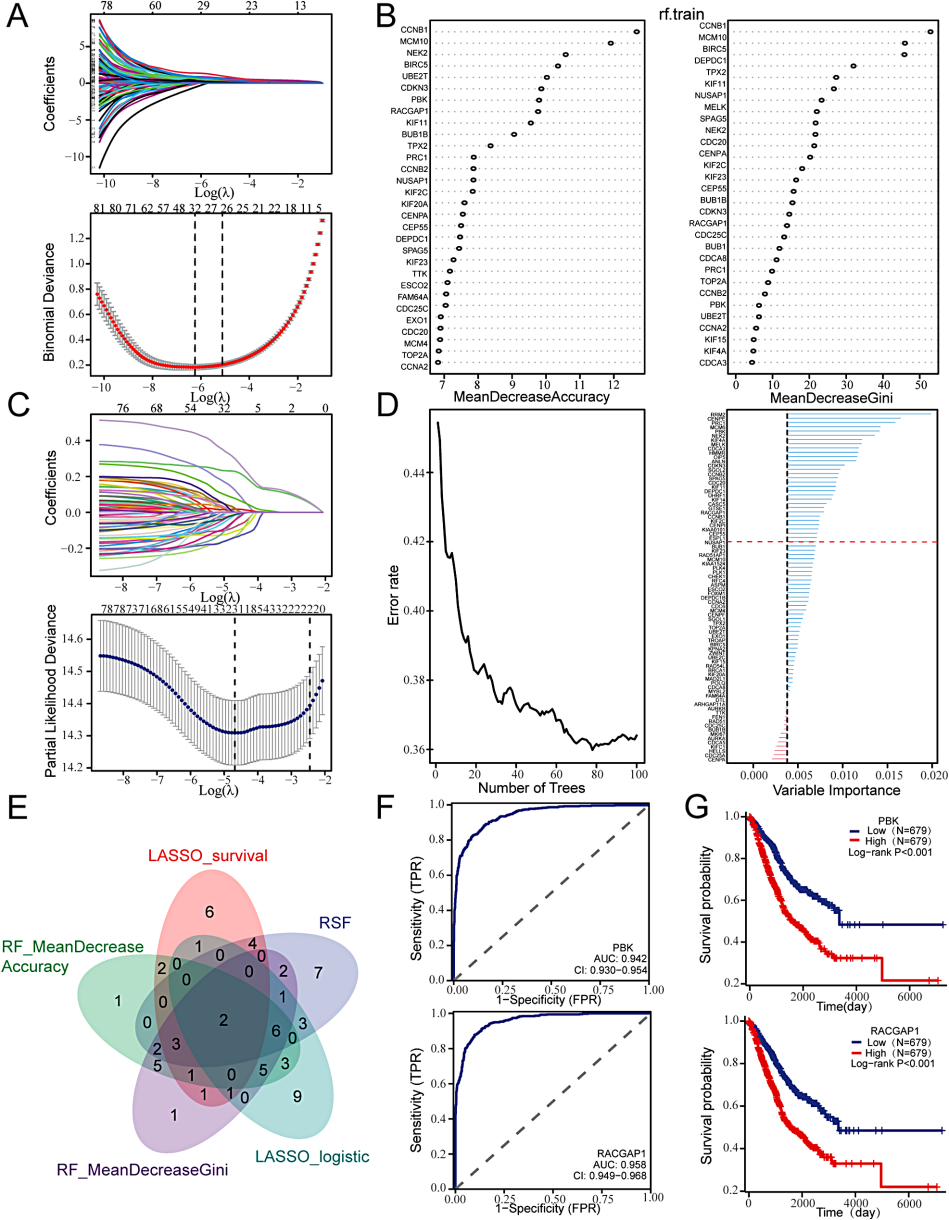
Machine learning was used to screen key genes. (**A**) Lasso regression analysis; (**B**) Random forest analysis; (**C**) Lasso-cox regression analysis; (**D**) Random survival forest analysis; (**E**) Venn diagram showing the overlap between the different machine learning methods; (**F**) The accuracy of different subtypes of stemness was evaluated by ROC curve; (**G**) Survival analysis of the high and low expression groups of PBK and RACGAP1.

We included 82 genes with the C2 subtype as the outcome variable. Firstly, a Lasso regression analysis was conducted on the merged LUAD dataset, revealing 31 genes significantly correlated with the C2 subtype (Fig. 3A). Subsequently, a random forest analysis was performed, and the top 30 genes were selected based on accuracy and Gini index weights (Fig. 3B).

For survival status as the outcome variable, the analysis included the same 82 genes. Lasso-Cox regression analysis on the merged LUAD dataset identified 21 genes (Fig. 3C), and the top 30 genes were selected using a random survival forest (Fig. 3D).

Finally, the intersection of genes selected by the four algorithms resulted in the identification of two hub genes: PBK and RACGAP1 (Fig. 3E).

Subsequently, the diagnostic performance of PBK (AUC: 0.942) and RACGAP1 (AUC: 0.958) in diagnosing the C2 subtype was demonstrated using ROC curves (Fig. 3F). Kaplan-Meier analysis showed that patients with high expression of PBK and RACGAP1 had significantly worse prognoses compared to those with low expression (Fig. 3G).

### RACGAP1 promotes proliferation and tumorigenicity of lung adenocarcinoma cells

Based on the previous research trends, there are many studies on PBK in non-small cell lung cancer [16–18]. However, there is little research on RACGAP1 in lung adenocarcinoma, so the role of RACGAP1 in lung adenocarcinoma is mainly studied.

**Fig. 4:**
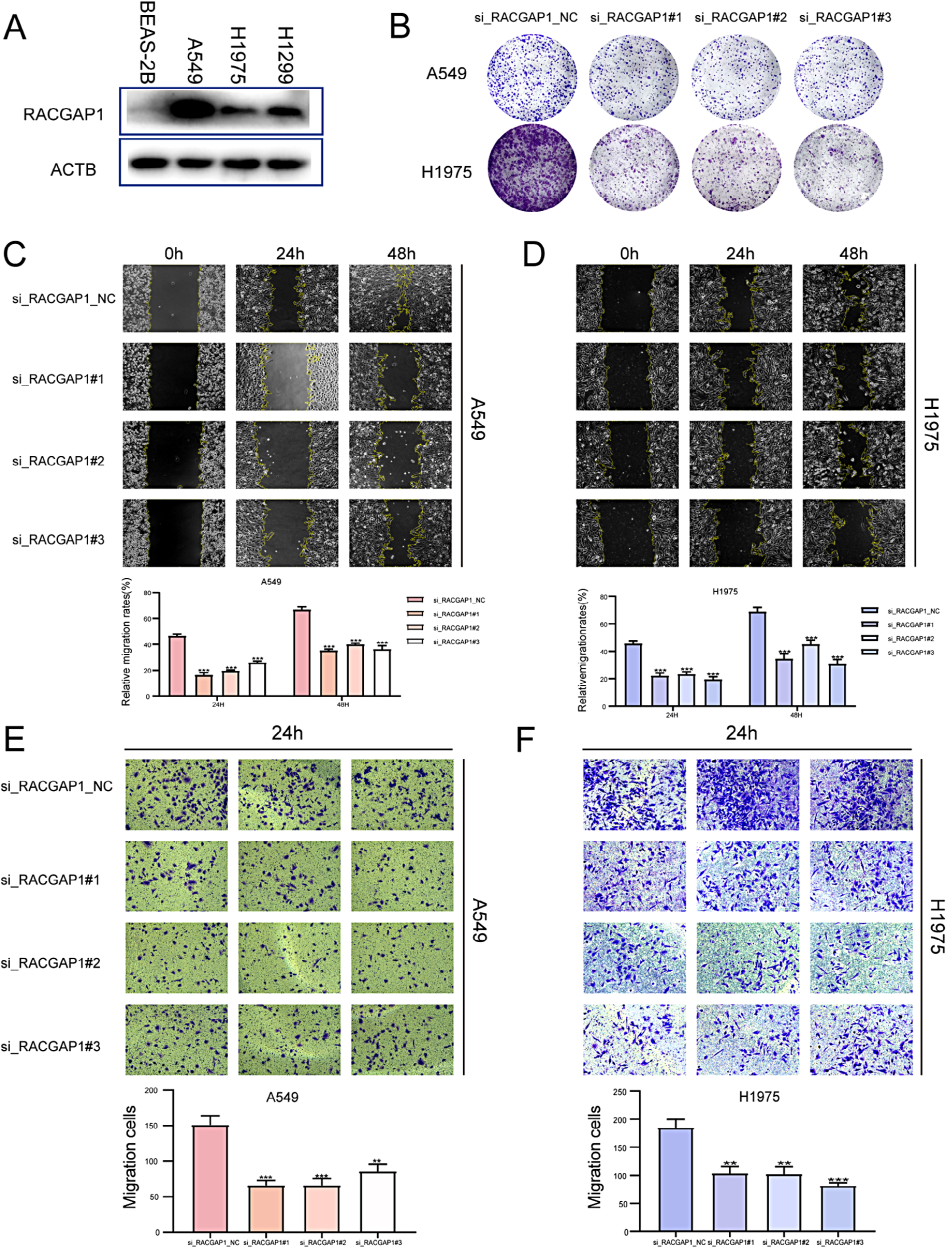
Cell phenotype experiments. (**A**) Western blot analysis was performed to determine the protein levels of RACGAP1 in four different lung cancer cell lines; (**B**) The cloning formation experiment was conducted after transfecting A549 and H1975 cells with si-RACGAP1; (**C**) A Wound healing assay was performed on A549 cells after transfection with si-RACGAP1; (**D**) A Wound healing assay was conducted on H1975 cells after transfection with si-RACGAP1; (**E**) Transwell migration assay was conducted on A549 cells after transfection with si-RACGAP1; (**F**) Transwell migration assay was performed on H1975 cells after transfection with si-RACGAP1.

The expression of RACGAP1 was examined using Western blot in both normal lung epithelial cell line BEAS-2B and three different lung adenocarcinoma cell lines (A549, H1975, H1299). The results showed that RACGAP1 was downregulated in H1975 cells and upregulated in A549 cells (Fig. 4A).

Subsequently, transient transfection using si-RACGAP1 was performed to knock down RACGAP1 in the two cell lines. The clonogenic formation assay revealed a significant inhibition of cell colony formation and an impact on cell proliferation upon RACGAP1 knockdown when compared to the negative control group (Fig. 4B).

The study also conducted cell scratch experiments (Fig. 4C-D) and transwell migration assays (Fig. 4E-F), which yielded consistent results. Knockdown of RACGAP1 significantly suppressed the migration ability of the cells.

### RACGAP1 regulation of the cell cycle in lung adenocarcinoma

**Fig. 5:**
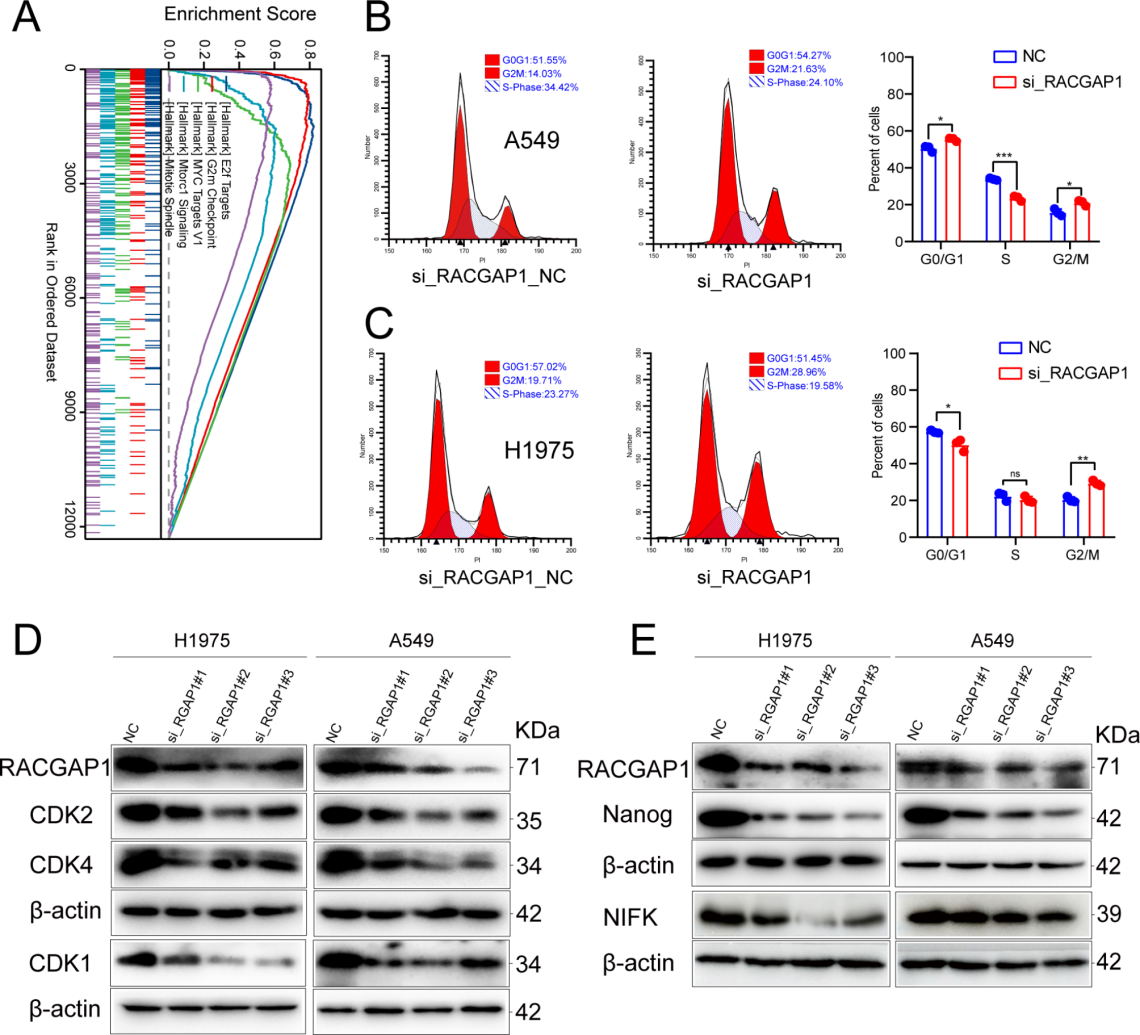
Flow cytometry analysis of cell cycle and WB validation of cell cycle protein and pluripotency-related protein expression. (**A**) GSEA analysis was performed on RACGAP1; (**B-C**) Flow cytometry analysis of cell cycle was conducted on A549 and H1975 cells after transfection with si-RACGAP1; (**D**) Compared to the negative control (NC), specific siRNA targeting RACGAP1 successfully inhibited the expression of RACGAP1 protein as well as cell cycle-related proteins CDK2, CDK4 and CDK1; (**E**) Western blot results showed that the expression of pluripotency-related markers NIFK and NANOG was downregulated after transfection with si-RACGAP1 (The blot has been cropped and the original blot is shown in the supplementary file.) (***p < 0.001; **p < 0.01; *p < 0.05; ns: not significant)

To further clarify the function of the RACGAP1 gene, GSEA analysis was conducted. Figure 5A displays the functional enrichment results for the top 5 genes. In the high-risk population, the RACGAP1 gene is primarily involved in encoding E2F transcription factors, which are related to cell cycle targets. It is also associated with oocyte meiosis, the G2/M checkpoint, genes regulated by MYC-version 1 (v1), and spindle assembly during mitosis.

To explore the role of RACGAP1 in the cell cycle, flow cytometry was performed, and the analysis showed that knocking down RACGAP1 arrested the cell cycle at the G2/M phase, affecting its progression (Fig. 5B-C).

To confirm the findings, Western blot analysis was conducted after knocking down RACGAP1. The specific siRNA successfully inhibited the expression of RACGAP1 protein as well as CDK2 and CDK4, two proteins associated with the cell cycle, compared to the negative control (NC) (Fig. 5D). Additionally, the expression of stem cell-related markers NIFK and NANOG was found to be downregulated during this analysis (Fig. 5E).

### Validation of RACGAP1’s role in tumors in vivo

**Fig. 6:**
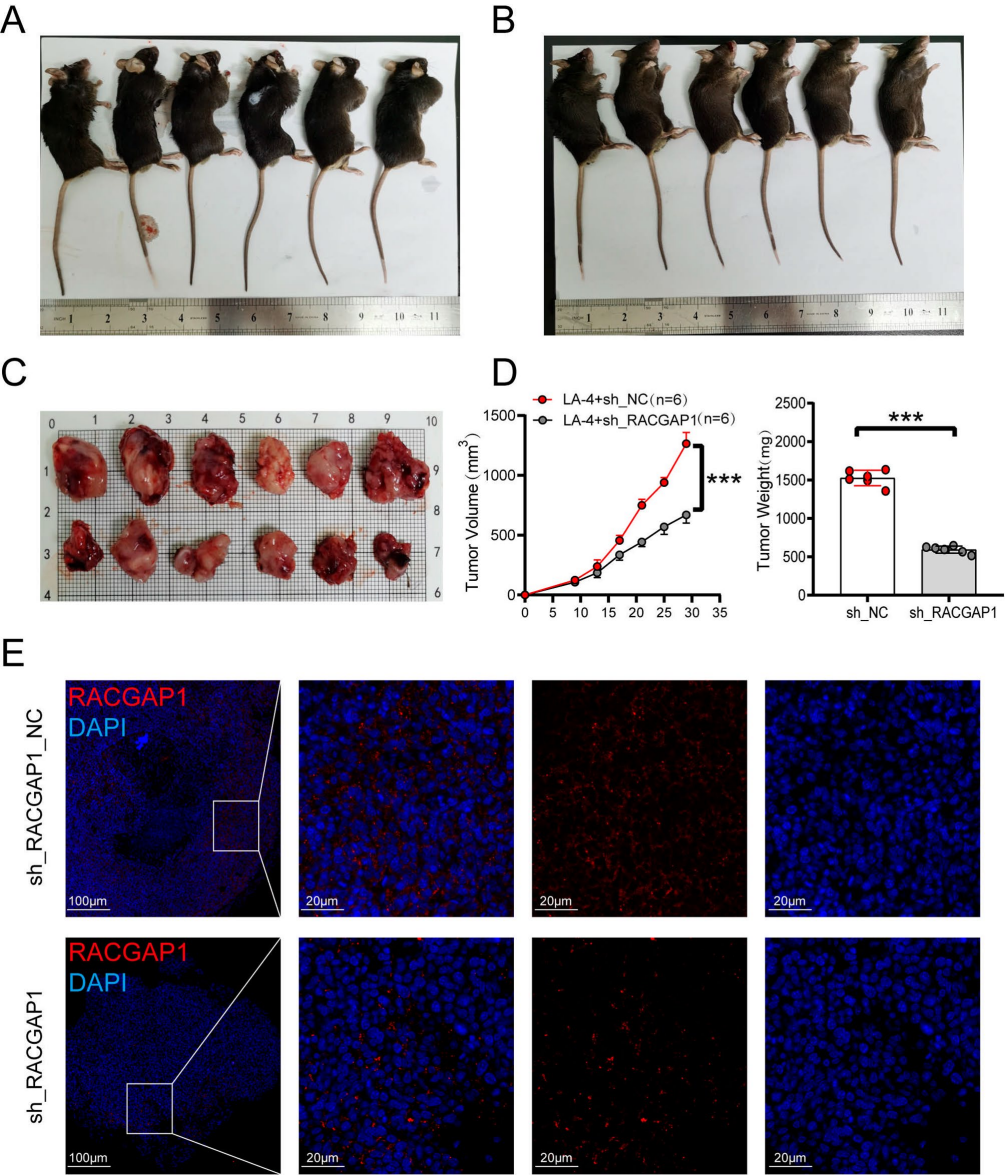
In vivo experimental validation. (**A-C**) Photographs showing euthanized mice and dissected tumors;(**D**) Line graph showing tumor volume and bar graph displaying tumor weight; (**E**) Ratio = 1 cm, immunofluorescence staining of tumor tissue and relative expression of RACGAP1 (* *P* < 0.05, ** *P* < 0.01, *** *P* < 0.001)

To accurately evaluate the role of RACGAP1 in tumors, RACGAP1 knockdown cell lines and control cells were subcutaneously injected into C57BL/6 mice. After a 30-day incubation period, the treatment of mice is shown in Fig. 6A-B, a significant reduction in tumor volume and weight was observed in the knockdown group compared to the control group (Fig. 6C-D). These findings were supported by immunofluorescence results, which showed a higher fluorescence signal in the negative control (NC) group compared to the knockdown group (Fig. 6E). These results provide further evidence of the close correlation between RACGAP1 expression and the occurrence and development of tumors.

## Discussion

In this study, genotyping through a set of 26 stemness genes identified two distinct stem cell-related subtypes, of which the C2 subtype was highly enriched in stemness phenotypic features and showed higher stemness score “mRNAsi”, lower sensitivity to immunotherapy, and poor prognosis. In order to accurately identify the C2 subtype, we screened two genes, PBK and RACGAP1, as biomarkers of stemness typing through comprehensive bioinformatics analysis, and the prognostic analysis showed that the prognosis of patients with LUAD was worse with the elevation of PBK and RACGAP1. We then verified that RACGAP1 could promote the tumor stemness phenotype and the rapid proliferation of tumor cells through cell phenotyping experiments and in vivo experiments in animals, which implies that RACGAP1 may serve as a potential therapeutic target for targeted stem cell therapy.

Patients with high expression of RACGAP1 in lung adenocarcinoma have a poorer prognosis, and Rac gTPase-activating protein 1 (RACGAP1) is a component of the central spindle complex, which acts as a microtubule-dependent and Rho-mediated signaling cytoplasmic divisions during the formation of myosin contractile rings [19]. It has been shown that RACGAP1 is overexpressed in a variety of cancers including breast [20], esophageal [21], gastric [22], hepatocellular carcinoma (HCC) [23], and ovarian [24], and that the high expression of RACGAP1 correlates with tumor aggressiveness and poor prognosis, which is in agreement with the present findings. By, cell scratch assay also further demonstrated that decreased RACGAP1 expression resulted in decreased lung cancer cell migration. In addition, RACGAP1 plays a role in regulating the cell cycle [25–27] and is involved in the progression of other types of cancer. For example, knockdown of RACGAP1 in the HCC cell lines SMMC7721 and HCCLM3 cells induces G2/M phase arrest and reduces cell number in G0/G1 phase [23]. In breast cancer, RACGAP1 promotes cancer cell metastasis by regulating mitochondrial mass thereby [28]. In this study, after reducing RACGAP1 expression in lung cancer cells A549 and H1975 by using si technique, flow cytometry revealed that the expression of cell cycle-related proteins CDK2 and CDK4 was reduced. Further in animal experiments, it was found that decreased RACGAP1 expression significantly reduced tumor growth, suggesting that RACGAP1 plays a non-negligible role in the progression of lung adenocarcinoma.

Tumor stem cells are a small subpopulation of cancer cells with the ability to self-renew and differentiate, and they are thought to play an important role in tumor maintenance and recurrence [29–33]. Numerous studies have shown that knockdown of the cell cycle-related proteins CDK1, CDK2, cyclin E or B1, or the use of CDK inhibitors results in the loss of pluripotency and the initiation of cell differentiation; In addition, studies of cyclin D or E-deficient mESCs have further confirmed that the cell cycle can regulate cell pluripotency [34–40]. In this study, the reduction of RACGAP1 expression was followed by western blot, which revealed that the stem cell biomarkers KI-67 and Nanog were similarly reduced in expression, suggesting a potential regulatory relationship between RACGAP1 and the tumor stemness phenotype. Therefore, we hypothesized that in lung adenocarcinoma, high expression of RACGAP1 leads to aberrant cell cycle regulation, which may include overactivation of cell cycle protein-dependent kinase (CDK) and deletion of CDK inhibitors, which prevents the tumor stem cells from being subjected to normal cell cycle regulation, thus increasing the activity of tumor cells.

However, there are still some limitations to our study. First, although multiple datasets have been used in this study and the datasets were processed with batch removal, potential bias may still exist. Second, the depth of research on RACGAP1 in this study is still lacking, and in-depth studies with the help of single-cell sequencing, gene editing, and other technologies are still needed.

## Conclusion

In this study, two stem cell-related subtypes with different prognoses, TME patterns and therapeutic responses were systematically identified for the first time by consistent clustering of stem cell gene sets. Through a series of bioinformatics, RACGAP1, a key stemness gene, was screened, and it was verified that RACGAP1 affects cell proliferation, migration and cell cycle regulation by cell phenotyping, in vivo experiments in animals, and flow cytometry analysis, thus affecting stemness, and thus it is expected to be used as a therapeutic direction and target for lung adenocarcinoma in the future.

### Electronic supplementary material

Below is the link to the electronic supplementary material.

Supplementary Material 1

## Data Availability

The original contributions presented in the study are publicly available. This data can be found here: https://www.cancer.gov/ccg/research/genome-sequencing/tcga(TCGA) and the datasets analyzed during the current study are available in the Gene Expression Omnibus (https://www.ncbi.nlm.nih.gov/geo/), including GSE13213, GSE26939, GSE72094, and GSE31210. The datasets used and/or analyzed in the current study are available from the corresponding author upon reasonable request. This study utilized datasets from the KEGG (https://www.genome.jp/kegg/) and GSEA (https://www.gsea-msigdb.org/gsea/index.jsp).

## References

[1] Yu QY (2022). CENPA regulates Tumor stemness in lung adenocarcinoma. Aging.

[2] Catalano V, et al. Tumor and its microenvironment: a synergistic interplay. Semin Cancer Biol. 2013;23(6 Pt B):522–32. 10.1016/j.semcancer.2013.08.007 24012661

[3] Batlle E, Clevers H (2017). Cancer stem cells revisited. Nat Med.

[4] Yu J (2018). Mechanistic exploration of Cancer Stem cell marker voltage-dependent Calcium Channel alpha2delta1 subunit-mediated Chemotherapy Resistance in Small-Cell Lung Cancer. Clin Cancer Res.

[5] Kuo MH (2020). Cross-talk between SOX2 and TGFbeta Signaling regulates EGFR-TKI Tolerance and Lung Cancer Dissemination. Cancer Res.

[6] Yu JJ (2020). TRIB3-EGFR interaction promotes Lung cancer progression and defines a therapeutic target. Nat Commun.

[7] Gettinger SN (2021). Nivolumab Plus Ipilimumab vs Nivolumab for previously treated patients with stage IV squamous cell Lung Cancer: the Lung-MAP S1400I phase 3 Randomized Clinical Trial. JAMA Oncol.

[8] Herbst RS (2021). Phase 1 expansion cohort of Ramucirumab Plus Pembrolizumab in Advanced Treatment-Naive NSCLC. J Thorac Oncol.

[9] Nogami N (2022). IMpower150 final exploratory analyses for Atezolizumab Plus Bevacizumab and Chemotherapy in Key NSCLC patient subgroups with EGFR mutations or metastases in the liver or brain. J Thorac Oncol.

[10] Owonikoko TK (2021). Nivolumab and Ipilimumab as maintenance therapy in extensive-disease small-cell Lung Cancer: CheckMate 451. J Clin Oncol.

[11] De Stefano L (2023). Tumor necrosis factor-alpha inhibitor-related autoimmune disorders. Autoimmun Rev.

[12] Mahdi J (2023). Tumor inflammation-associated neurotoxicity. Nat Med.

[13] Kanehisa M, Goto S (2000). KEGG: kyoto encyclopedia of genes and genomes. Nucleic Acids Res.

[14] Kanehisa M (2019). Toward understanding the origin and evolution of cellular organisms. Protein Sci.

[15] Kanehisa M (2023). KEGG for taxonomy-based analysis of pathways and genomes. Nucleic Acids Res.

[16] Li J, Hou W (2021). Expression patterns and clinical significances of PBK in Lung cancer: an analysis based on Oncomine database. Transl Cancer Res.

[17] Wang P (2023). UPF1 regulates FOXO1 protein expression by promoting PBK transcription in non-small cell Lung cancer. Biochem Biophys Res Commun.

[18] Shih MC (2012). TOPK/PBK promotes cell migration via modulation of the PI3K/PTEN/AKT pathway and is associated with poor prognosis in Lung cancer. Oncogene.

[19] Wang MY (2019). Pseudogene RACGAP1P activates RACGAP1/Rho/ERK signalling axis as a competing endogenous RNA to promote hepatocellular carcinoma early recurrence. Cell Death Dis.

[20] Pliarchopoulou K (2013). Prognostic significance of RACGAP1 mRNA expression in high-risk early Breast cancer: a study in primary tumors of Breast cancer patients participating in a randomized Hellenic Cooperative Oncology Group trial. Cancer Chemother Pharmacol.

[21] Zhao W (2020). RACGAP1 is transcriptionally regulated by E2F3, and its depletion leads to mitotic catastrophe in esophageal squamous cell carcinoma. Ann Transl Med.

[22] Saigusa S (2015). Clinical significance of RacGAP1 expression at the invasive front of gastric cancer. Gastric Cancer.

[23] Yang XM (2018). Overexpression of rac GTPase activating protein 1 contributes to proliferation of Cancer cells by reducing Hippo Signaling to Promote Cytokinesis. Gastroenterology.

[24] Wang C (2018). Rac GTPase activating protein 1 promotes oncogenic progression of epithelial Ovarian cancer. Cancer Sci.

[25] Warga RM (2016). Progressive loss of RacGAP1/ogre activity has sequential effects on cytokinesis and zebrafish development. Dev Biol.

[26] Schneid S (2021). The BRCT domains of ECT2 have distinct functions during cytokinesis. Cell Rep.

[27] Kim H (2014). Centralspindlin assembly and 2 phosphorylations on MgcRacGAP by Polo-like kinase 1 initiate Ect2 binding in early cytokinesis. Cell Cycle.

[28] Zhou D (2021). Long non-coding RNA RACGAP1P promotes Breast cancer invasion and Metastasis via miR-345-5p/RACGAP1-mediated mitochondrial fission. Mol Oncol.

[29] Uxa S (2021). Ki-67 gene expression. Cell Death Differ.

[30] Heurtier V (2019). The molecular logic of nanog-induced self-renewal in mouse embryonic stem cells. Nat Commun.

[31] Wolff SC (2018). Inheritance of OCT4 predetermines fate choice in human embryonic stem cells. Mol Syst Biol.

[32] Kunihiro AG (2022). CD133 as a biomarker for an autoantibody-to-ImmunoPET paradigm for the early detection of small cell Lung Cancer. J Nucl Med.

[33] Chen C (2018). The biology and role of CD44 in cancer progression: therapeutic implications. J Hematol Oncol.

[34] Coronado D (2013). A short G1 phase is an intrinsic determinant of naive embryonic stem cell pluripotency. Stem Cell Res.

[35] Wang XQ (2017). CDK1-PDK1-PI3K/Akt signaling pathway regulates embryonic and induced pluripotency. Cell Death Differ.

[36] Neganova I (2009). Expression and functional analysis of G1 to S regulatory components reveals an important role for CDK2 in cell cycle regulation in human embryonic stem cells. Oncogene.

[37] Filipczyk AA (2007). Differentiation is coupled to changes in the cell cycle regulatory apparatus of human embryonic stem cells. Stem Cell Res.

[38] Gonzales KA (2015). Deterministic restriction on pluripotent state dissolution by cell-cycle pathways. Cell.

[39] Zhang WW (2011). Cdk1 is required for the self-renewal of mouse embryonic stem cells. J Cell Biochem.

[40] Neganova I (2014). CDK1 plays an important role in the maintenance of pluripotency and genomic stability in human pluripotent stem cells. Cell Death Dis.

