# Supplementary figures and images for: RACGAP1 promotes the progression and poor prognosis of lung adenocarcinoma through its effects on the cell cycle and tumor stemness
Source: BMC Cancer. 2024 Jan 2;24:7. doi: 10.1186/s12885-023-11761-x (PMC10763365; doi:10.1186/s12885-023-11761-x)

Figure 4A

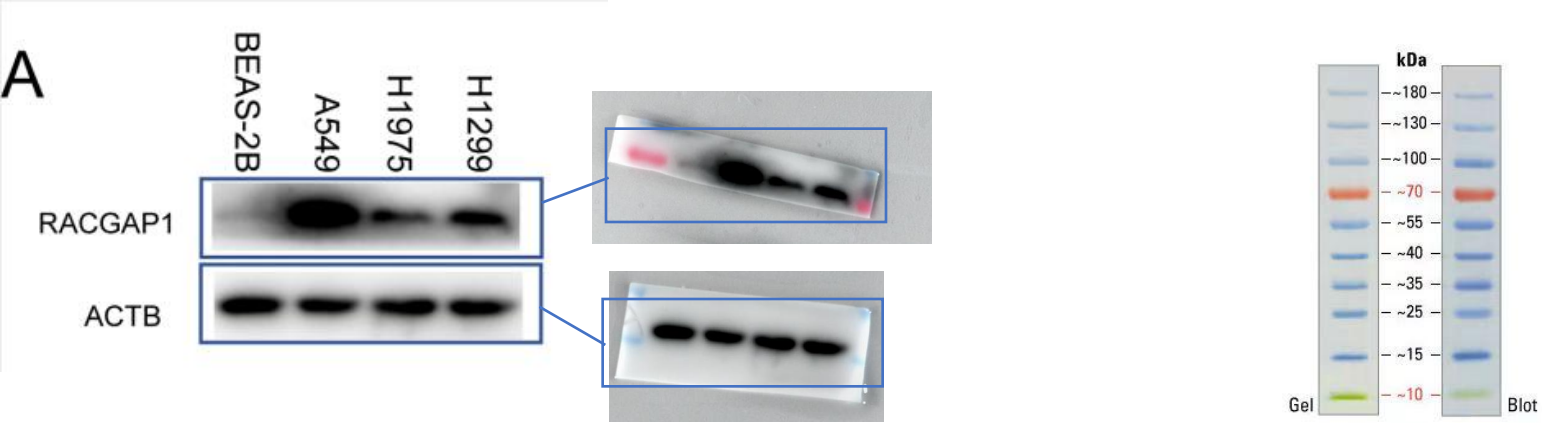

Figure 5D

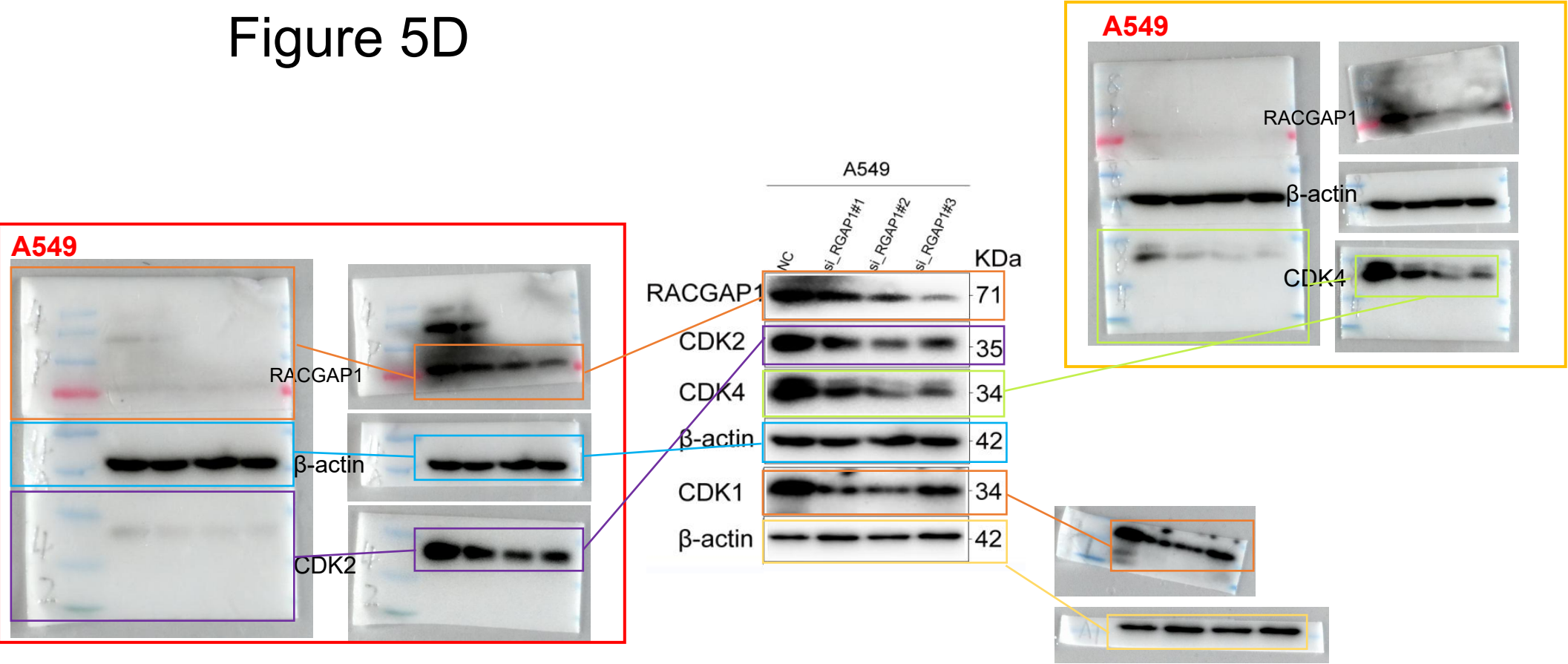

# Figure 5D

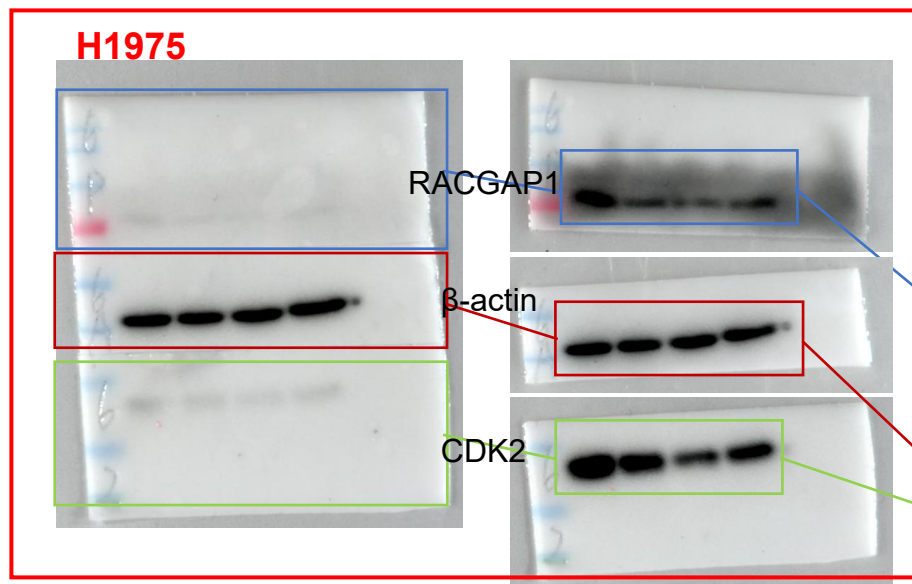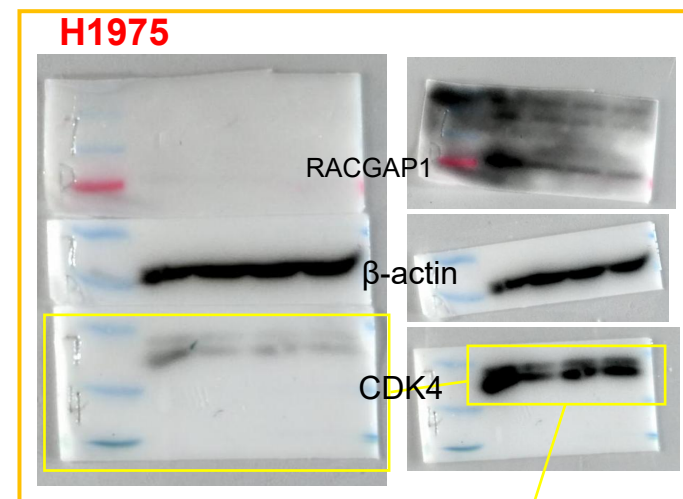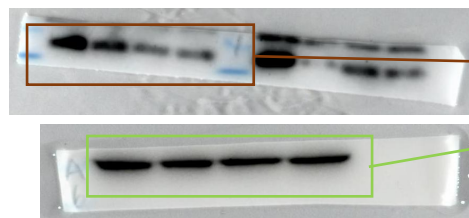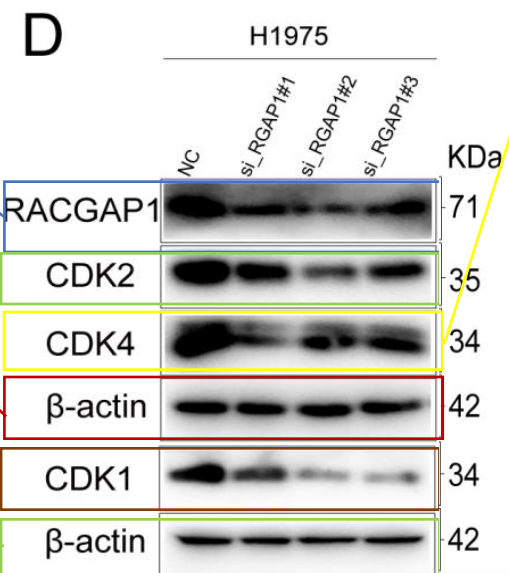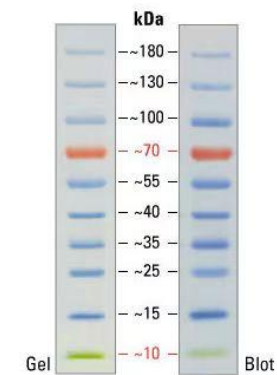

Figure 5E

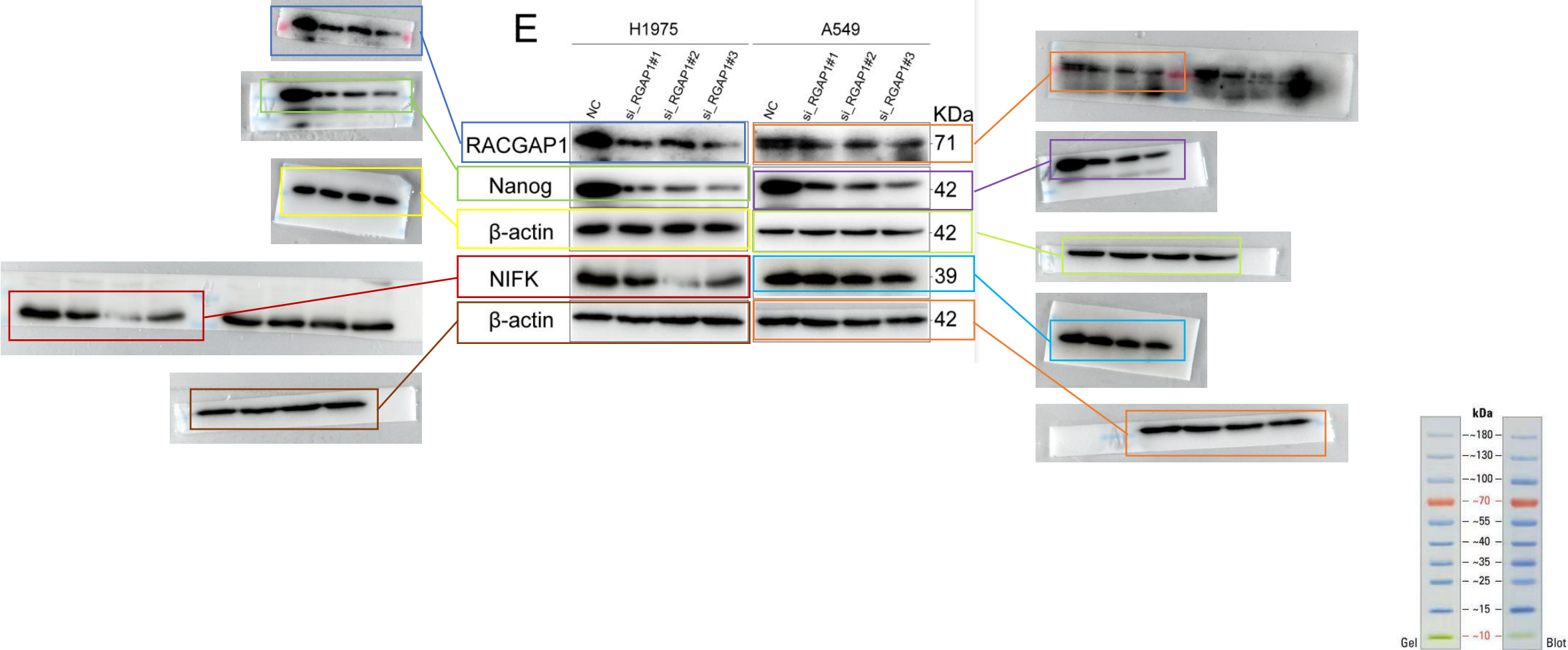

Supplement: Supplementary file 1 — Supplementary Material 1 [file 12885_2023_11761_MOESM1_ESM.pdf]
